# Computational Approaches to Music Motor Performance: Clustering of Percussion Kinematics Underlying Performance Style

**DOI:** 10.3389/fpsyg.2021.725016

**Published:** 2021-12-16

**Authors:** Tristan Loria, Aiyun Huang, Tara Lynn Henechowicz, Michael H. Thaut

**Affiliations:** ^1^Music and Health Research Collaboratory, Faculty of Music, University of Toronto, Toronto, ON, Canada; ^2^Faculty of Music, University of Toronto, Toronto, ON, Canada

**Keywords:** motor control, kinematics, music performance, percussion, sensorimotor, performance style

## Abstract

The present study investigated motor kinematics underlying performance-related movements in marimba performance. Participants played a marimba while motion capture equipment tracked movements of the torso, shoulders, elbows, wrists, and hands. Principal components analysis was applied to assess the movements during the performance related to sound production and sound preparation. Subsequent cluster analyses sought to identify coupling of limb segment movements that may best characterize performance styles present in the performance. The analysis revealed four clusters that were thought to reflect performance styles of expressive performance, postural sway, energy efficiency, and a blend of the former styles. More specifically, the expressive cluster was best characterized by limb movements occurring along the vertical z-axis, whereas the postural sway cluster was characterized by forwards and backwards motions of the torso and upper limbs. The energy efficient cluster was characterized by movements of the body moving left to right along the marimba, whereas the blended style demonstrated limited delineation from the alternate styles. Such findings were interpreted as evidence that performance styles occur within a framework of biomechanical constraints and hierarchical stylistic factors. Overall, the results provided a more holistic understanding of motor execution in percussion performance.

## Introduction

Musicians, such as percussionists, execute thousands of instrumental gestures during a performance (i.e., here referred to as sound-producing movements). For a percussion instrument like the marimba, sound production is the product of precisely controlled movements of the torso, shoulders, elbows, wrists, and hands. However, motor control strategies underlying sound production in marimba performance remain poorly understood. Further advancing knowledge of marimba performance from a kinematic perspective is indeed relevant to the performance science and complex action execution literature. Regarding complex action execution, marimba performance involves supination/pronation, flexion/extension, and rotation of the upper limbs which has considerable overlap with non-musical complex motor tasks such as sports (e.g., baseball). Regarding performance science, precisely identifying the motor control characteristics associated with marimba performance is expected to advance fundamental knowledge of sensorimotor control in music in general and percussion specifically. Doing so would facilitate empirical understanding of motor control in music, as well as potentially advance theories of how musical motor skills may best be acquired. Therefore, the focus of the present study was to further elucidate sound-producing movement execution in marimba performance from a kinematic perspective.

Studies investigating percussion kinematics have revealed how sound-producing movements of a drumstick and hand are executed along the vertical movement axis (i.e., *z*-axis). In percussion, movements of the upper limbs are aimed at sound production, and thus movement amplitude and sound characteristics are closely linked. Dahl (2000) reported that strokes at higher dynamic levels like forte were initiated from a greater height above the instrument than softer strokes, thus demonstrating a clear link between stick motion and sound. The vertical displacement of the drumstick was also greater in preparation for an accented relative to unaccented stroke; however, this vertical displacement was reduced when playing at faster tempi (i.e., Dahl, 2004, 2011; see also: Furuya and Kinoshita, 2008 for a piano context). From a motoric perspective, sound production likely results from the interplay between movement kinematics and the desired musical outcome.

Recent work investigating experienced vs. inexperienced drummers has shown that expert performance was more fluent, efficient, and accurate compared to lesser skilled counterparts. For example, expert drummers were more temporally accurate in drumming strokes than trainees and non-musicians, a finding driven by whiplash-like movements of the wrists and hands along the vertical *z*-axis (i.e., Altenmüller et al., 2020). Beveridge et al. (2020) reported that expert drummers engaged wrist flexor muscles more so than the extensor muscles, while amateurs tended to engage flexor and extensor muscles equally. Such findings may contribute to the overall smoothness, or fluency, in which experts execute sound-producing movements (see Gonzalez-Sanchez et al., 2019). However, such findings shed limited light on how limb movement patterns of proximal and distal limb segments (i.e., shoulders, elbows, and hands) are organized from a stylistic perspective to produce sound.

Sound-producing movements in percussion are complex counterparts of typical reaching movements. Visual feedback of the moving limb is critical to control movements online during activities of daily living (i.e., Meyer et al., 1988; Sober and Sabes, 2005; Elliott et al., 2010, 2017). However, the sequential and rapid execution of musical movements reduces the ability to utilize feedback of the limb’s trajectory and thus, musical movements can be considered ballistic and reliant on motor planning mechanisms (e.g., Keele, 1968; Beggs and Howarth, 1970; Ghez et al., 1997). Motor planning in percussion contexts like marimba performance may be particularly challenging when considering the role of biomechanics in sound production and issues related to movement redundancy.

Sound production and the associated ancillary gestures can result from an almost infinite combination of actions. Indeed, biomechanical factors, such as the extensive range of motion for flexion/extension and pronation/supination of the elbows and wrists (e.g., Norkin and White, 2009; Clarkson, 2013), subsequently impact bodily movements underlying sound production (e.g., Bejjani and Halpern, 1989; Wanderley et al., 2005). Such constraints are further emphasized when considering the redundancy problem, or the issue of selecting the most appropriate motor commands among an enormous pool of alternative actions (e.g., Wolpert, 1997; Flash et al., 2013). Marimba is a staccato instrument wherein once an individual bar/note is struck, the sound decays rapidly (e.g., Fletcher and Rossing, 1998). As a result, performers often manipulate upper limb movements in the form of executing stylized gestures (i.e., performance styles) to influence the perceived aural quality of the performance (e.g., Schutz and Lipscomb, 2007; Broughton and Stevens, 2009; Jensenius and Wanderley, 2009). Therefore, marimba performance offers a novel perspective to examine the redundancy problem, given that performers must balance biomechanical constraints with stylistic performance features that are not present in non-melodic percussion contexts such as snare drumming.

Marimba performance may be ideally suited to address how biomechanical constraints are interpreted and executed in a percussion context. Indeed, utilizing a marimba-based paradigm permits the investigation of execution mechanisms in music where the *x* (i.e., moving mediolaterally from left to right) and *y* (i.e., moving anteroposterior from front to back) cartesian planes are relevant, which compliments previous investigations focused on the *z* cartesian plane (i.e., moving vertically, see Dahl, 2011; Altenmüller et al., 2020). In addition, the qualitative interpretation of marimba performance movements (i.e., Broughton and Stevens, 2009; Broughton and Davidson, 2016) can be strengthened by objectively examining performance styles from a movement kinematics perspective. Lastly, the number of potential styles that may exist in a group of percussionists playing the marimba remains undetermined. Developing such knowledge is expected to yield a more holistic understanding of percussion motor control.

A computational clustering approach (i.e., unsupervised machine learning) was utilized to examine kinematic-based styles present within a musical excerpt performed by trained percussionists. Principal components analysis (PCA) was used as a tool that began with assessing whole upper limb movements (e.g., Federolf et al., 2014; Massie-Laberge et al., 2019). After the PCA was applied on the matrix of kinematic data, clustering analysis of individual PCA scores was employed to objectively determine the number of potential performance styles, and identify the underlying limb segments that best characterized the sound-producing movements for each style. Given the redundancy problem raised above, movement pattern clustering may provide an objective approach for assessing performer style and to further understand how proximal and distal limb segments interact during a performance. Developing such an understanding could provide novel insight into kinematic characteristics present in marimba performance that may be leveraged in the future to enhance motor learning in percussion.

## Materials and Methods

### Participants

Demographic information of the 15 participants (# of female participants = 10, # of male participants = 5) who voluntarily completed the experimental protocol is found in Table 1. Three professional performers (i.e., two faculty members) and twelve students in the Faculty of Music at the University of Toronto participated. Participants self-reported to having an average of 14.5 years (SD = 3.9) of formal music education and 10.5 years (SD = 6.3) of percussion specific training. In addition, participants estimated they were practicing 21.5 h per week (SD = 10.1) at the time of testing. The study was approved by the University of Toronto Research Ethics Board and participants provided informed written consent.

**Table 1:** Demographic information including the amount of formal and percussion specific music education, and time spent practicing is shown for all participants.

Participant	Formal music education (years)	Percussion specific training (years)	Hours of practice/week
P1	14	28	6
P2	18	15	28
P3	24	18	12
P4	16	8	16
P5	10	6	35
P6	8	8	26
P7	16	8	16
P8	10	6	35
P9	12	12	12
P10	16	8	25
P11	14	1	14
P12	18	12	38
P13	14	8	30
P14	14	10	12
P15	14	9	18

### Apparatus

**Figure 1:**
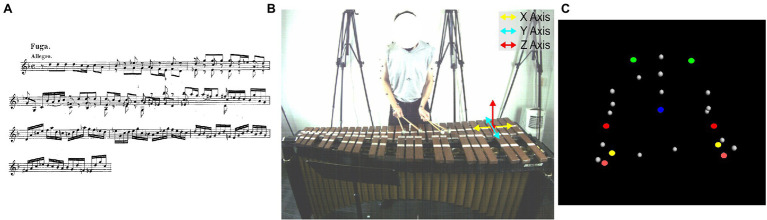
The performance excerpt **(A)**, movement axes **(B)**, and marker placements **(C)**. For the marker placements, the torso maker is colored blue, the shoulders green, the elbows red, the wrists yellow, and the hands in light pink.

An excerpt comprised of the first 10 measures from the second movement of J.S. Bach’s Sonata No.1 in G minor was performed on a Musser Deluxe Studio Grand Rosewood M245 marimba (Ludwig Musser, Elkhart, Indiana, United States). The piece was selected because it had no tempo changes and participants were instructed to ignore changes in dynamics (see Figure 1). Participants played the excerpt at 72 beats-per-minute (bpm) as indicated by an auditory metronome using two mallets in each hand. This tempo resulted in a performance that was 33 s long for each participant in the experiment.

**Figure 2:**
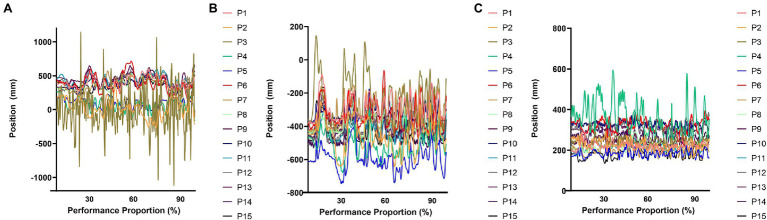
Individual participant data pertaining to movements of the right elbow throughout the performance is shown. Movements occurring along the X, Y, and Z axes are shown in **(A)**, **(B)**, and **(C)**, respectively.

Motion capture technology was used to gather kinematic data of limbs and torso movements. The setup included eight Vicon Vero version 2.2 (Vicon Motion Capture, Oxford, United Kingdom) high speed motion capture cameras. The system’s standard resolution is 2.2 megapixels (i.e., 2048 × 1088) with a camera latency of 3.6 ms and spatial resolution of 0.1 mm. In total, 27 markers were affixed to the limbs of the performers (see Figure 2) in line with the upper limb model written in Vicon BodyLanguage (e.g., Murray, 1999). Five markers were positioned on the upper half of the torso including the spinous process of the seventh cervical vertebra, the right scapula, the spinous process of the tenth thoracic vertebra, the jugular notch where the clavicles meet the sternum, and the xiphoid process of the sternum. The marker positioned on the xiphoid process of the sternum was used to track movements of the torso. Markers were further positioned on the left and right limbs including on the acromion-clavicular joint (i.e., used to measure movements of the shoulders), upper arms, the lateral epicondyle approximately at the elbow joints (i.e., used to measure movements of the elbows), the midpoint of the forearms, the thumb side of the radial styloid (i.e., used to measure wrist movements), the little finger side of the ulnar styloid, and just below the third metacarpus on both hands (used to measure hand movements, see Murray, 1999; Cutti et al., 2005). The position of the markers were recorded at a sampling frequency of 100 Hz.

To facilitate the application of this work into the training of percussionists, joint position/movement analyses were prioritized over joint angles (i.e., limb orientation). That is, focusing on joint movements may result in targeted motor learning feedback which could take the form of verbal cues that instructors could provide during training (i.e., bring your elbows closer to the playing surface). In contrast, orientation data may be more challenging to implement *via* targeted verbal cues in pedagogical contexts (i.e., maintain a certain elbow flexion angle). It is expected that providing this initial study will encourage additional inquiry into how performance style influences limb orientation, which is expected to further understanding of sensorimotor control in percussion performance.

### Procedure

Participants were provided with the excerpt *via* e-mail one week prior to participation for familiarization purposes. The goal during collection was to record a performance that best exemplified the participant’s musical ability. After a trial, the experimenter asked the performer if that trial represented their ability. The trial indicated by the performer as meeting this criterion was saved for data analysis. That is, one trial was saved from each participant and used for formal data analysis.

### Data Analysis

Data were recorded along three movement axes (see Figure 2). The *x*-axis ran from medial to lateral, or from left to right, along the marimba. Movements that occurred to the right of the origin were positive and movements to left of the origin were negative. The *y*-axis ran anterior to posterior, or forwards and backwards, along the marimba and shared the same origin position as the *x*-axis. This origin was set to the position of the resonator below the central bar of the marimba. Movements above the resonators were positive and movements below the resonators were negative. Movements were measured from the superior to inferior along the *z*-axis (i.e., vertical). The limb segments of interest included the torso, shoulders, elbows, wrists, and hands. Prior to analyses, all data were bidirectionally processed using a low-pass fourth order zero-lag Butterworth filter (i.e., Butterworth, 1930) with a cut-off frequency of 6 Hz.

#### Principal Components Analysis

Each participant had a time series matrix with 3,310 time points (i.e., rows) for 27 limb position variables (e.g., left elbow x-axis and right elbow x-axis). The 15 participant matrices were concatenated into a single matrix creating a matrix with 3,310 time points × 15 participants, yielding a total of 49,650 rows of data. Of these data points, 37 rows were removed for missing data, which resulted in a final matrix for the PCA of 49,613 observations of 27 limb segment movements. The data were both centered to a mean of 0 and scaled to have one unit of variance variance prior to conducting the PCA (i.e., for a similar application see: Massie-Laberge et al., 2019).

PCA was then conducted using R version 3.6.1 (R Studio Team, 2015). PCA is an unsupervised machine learning technique that computes an orthogonal set of variables to account for a large portion of variability in the original dataset. PCA reduces the dimensions of a dataset using linear algebra to reorganize the original data into components that explain a certain proportion of total variance. The proportion of variance explained by each component determines the ranking of the components, with principal component (PC) 1 explaining the greatest amount of variance followed by the remaining principal components. PCs were obtained by contrasting the variables in the covariance matrix using single value decomposition (see Jolliffe, 2002). The PCA was used to systemically assess movements of the upper limbs and torso across all three movement axes. The resulting PCs represent limb segment movements (e.g., left wrist) and directions (e.g., mediolateral), wherein the relationship between movements of each limb segments was further examined using an objective clustering approach.

#### Clustering of PCA Results

Kmeans clustering was subsequently applied on the rotated individual data for all 27 components. The seed was set to 150 and the number of clusters was determined *apriori*. This was accomplished using an elbow plot of the number of clusters derived from the within group sum of squares to determine the point of inflection. Kmeans clustering was conducted using the Euclidean distance metric, with the predetermined optimal k-value, 10 iterations, and 50 random sets of centers. The clusters of individual data were plotted in three dimensions on the first three principal components which accounted for 65% of the cumulative variance. Biplots of the individual PCA scores and the variable loadings were plotted to ascertain the presence of performer style.

## Results

### Principal Components Analysis

**Figure 3:**
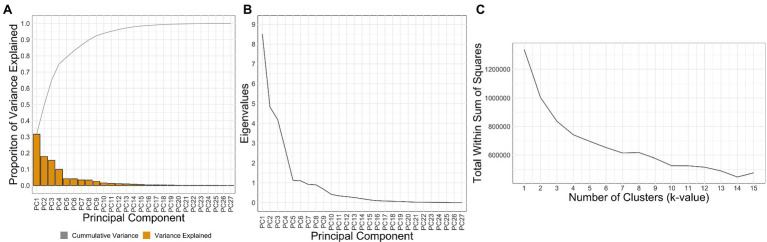
The cumulative and individual variance explained by each PC is shown in **(A)**, the eigenvalues associated with each PC in **(B)**, and an elbow plot used to determine the number of clusters present in the data in **(C)**.

The first 6 PCs had an eigenvector >1, while also explaining 83% of the cumulative variance. The explained variance and cumulative variance for all PCs are shown in Figure 3. The analysis showed the following standard deviation values associated with each PC: PC1 = 2.9 mm, PC2 = 2.2 mm, PC3 = 2.1 mm, PC4 = 1.6 mm, PC5 = 1.1, and PC6 = 1.1 mm. Individual PC scores can be found in Supplementary Material.

### Clustering Results

**Figure 4:**
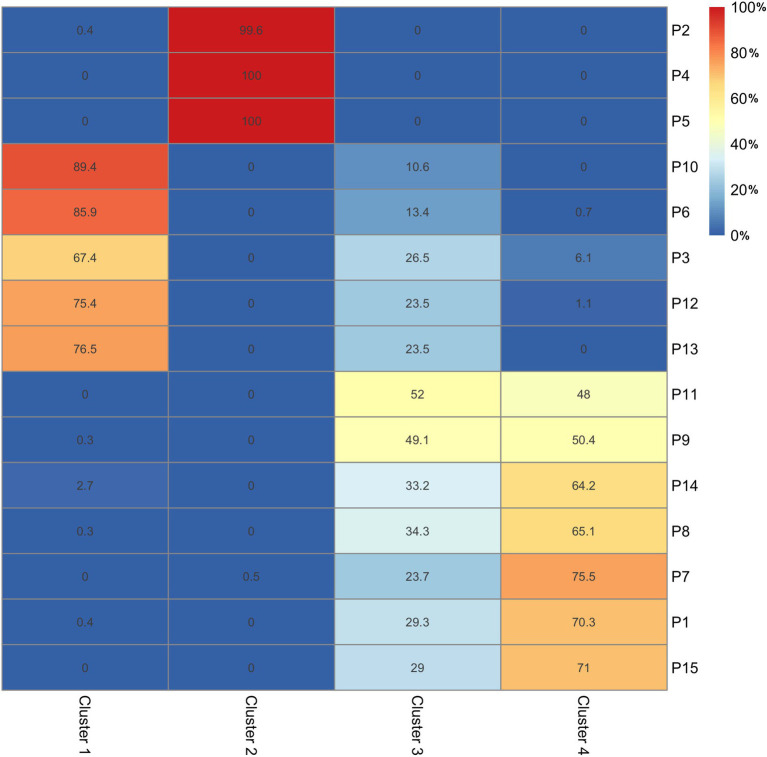
A heat map representing the clustering of kinematic data in the analysis. Values are expressed as a percentage of the kinematic data belonging to a specific cluster. The heat map values range from 0 (i.e., blue) to 100% (i.e., red). The extracted clusters are shown along the x-axis and the participant’s belonging to said cluster is displayed along the y-axis.

Based on the PCA, four optimal clusters were identified, each with sizes of 13,190, 11,522, 9,929, and 14,972 samples. The cluster analysis revealed that four different types of movement strategies (i.e., styles) with unique kinematic characteristics best represented the variance in the total dataset. The participant membership (i.e., expressed as a percentage of the participants’ kinematic data) within each cluster is shown in Figure 4.

**Figure 5:**
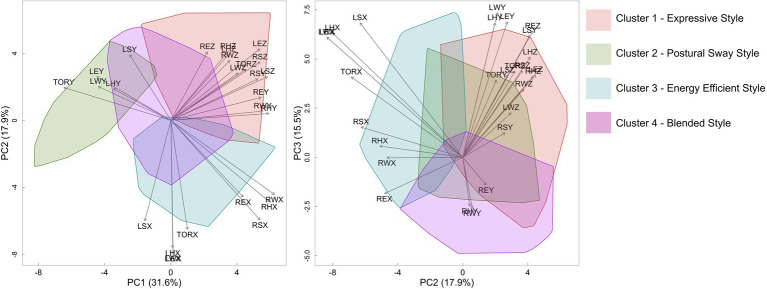
Eigenvectors (in gray) show how strongly each variable influences the principal components. Polygons represent the shape of the clusters of individual data projected back onto the principal components.

Performance styles were extracted and inferred from the clustering of kinematic data shown in Figure 5 (see also Supplementary Material). Analysis of cluster 1 (i.e., the red cluster in Figure 5) shows individual data that is in the top right quadrant, corresponding to positive loadings associated with the entire left and right limb along the z-axis, and right limb along the y-axis. Given the loadings for both the upper limbs, the style demonstrated by participants in this cluster (i.e., P10, P6, P3, P12, and P13) can best be characterized by vertical movements of the upper limbs along the z-axis during sound production. This cluster may be considered consistent with an expressive performance style.

The analysis revealed that cluster 2 was associated with unique movement strategies relative to the remaining clusters of kinematic data. When the individual data were projected onto PC1 and PC2, participants in cluster 2 (i.e., as shown in green in Figure 5), are distributed in the upper left quadrant with large magnitude scores onto PC1. Further examination of the eigen vectors revealed that scores in the upper left quadrant comprised movements of the left shoulder, elbow, wrist, and hand as well as the torso along the y-axis (i.e., anteroposterior, moving forwards and backwards above the instrument). Based on this cluster and the resulting limb movements associated with it, it may be surmised that participants (i.e., P2, P4, and P5) in this cluster have a unique style wherein movements of the torso and left limb work in tandem during the performance. As a result, a postural sway style was considered to reflect the style associated with cluster 2.

As shown in Figure 5, the blue cluster representing cluster 3 distributed on the positive regions of PC1, negative regions of PC2, and positive regions of PC3, with loadings for the left and right limbs (i.e., elbows, hands, wrists, and shoulders) and torso along the x-axis (i.e., moving left to right along the instrument). When viewed in conjunction with the alternate clusters, it may be surmised that performers in cluster 3 focused on movement efficiency during the performance. More specifically, because movement patterns were predominantly identified along the x-axis, it may be suggested that performers simply moved their limbs and torso into a position to strike the bars in the most efficient and direct manner possible. As a result, cluster 3 may represent an energy efficient playing style.

Cluster 4, wherein scores are in the bottom right quadrant, had large loadings for the right elbow, wrist, and hand along the y axis (i.e., positive on PC2, negative on PC3). It is worth noting that cluster 4 shares considerable overlap with the remaining clusters and is most often fixed to the center of the eigenvector plots shown in Figure 5. It may be surmised that cluster 4 represents a style that is not clearly delineated from the previously identified clusters. Therefore, the style that best characterizes movement execution in cluster 4 can be considered as a blend, or a mix of the other three styles. This hypothesis was further discussed below.

## Discussion

This study evaluated whether distinct styles could be extracted from upper limb motion patterns in marimba performance to shed further light on motor control in percussion. Sound-producing movements were investigated by employing PCA combined with cluster analysis to identify potentially unique and common styles that best characterized kinematic approaches to sound production. This analysis indicated the presence of four unique clusters based on movement patterns. The clusters were surmised to reflect differing performance styles present in the data set including expressive (i.e., cluster 1), postural sway (i.e., cluster 2), energy efficient (i.e., cluster 3), and blended (i.e., cluster 4) styles. To the best of our knowledge, this is the first study attempting to objectively quantify performance styles in marimba performance using computational approaches. The implications of the present findings regarding performance styles were considered below in line with previous literature, biomechanical findings, and theoretical proposals.

The expressive style associated with cluster 1 was consistent with previous work on percussion kinematics. This expressive style was characterized by movements of both limbs along the vertical z-axis. This finding agrees with previous findings for snare drumming regarding the vertical displacement of the drumstick and the subsequent impact on sound characteristics. For example, Dahl (2000) has previously shown that expressive gestures including greater vertical displacement of the drumstick coincided with playing accents and that such vertical displacement was reduced at fast vs. slow tempi (i.e., Dahl, 2004). Displacement of the upper limbs along the vertical z-axis has further been shown to differentiate expert vs. trainee drummers (e.g., Altenmüller et al., 2020), suggesting that upper limb movements are a critical and stable motion pattern in percussion. However, the novel contribution of the present study is the fact that multiple clusters (i.e., styles) were present which suggests that the stylistic properties of sound production reported in previous work may exists in parallel with alternate performance styles revealed here.

Perhaps the most unique style identified in the present analysis was a postural sway approach. The postural sway cluster was characterized by movements of the left limb and torso along the y-axis. In other words, torso movements may best be characterized by a swaying back and forth motion executed by these performers (see also Nusseck and Wanderley, 2009). Movements of the torso including swaying is well documented, and often leveraged, in musical and rhythmic contexts. In piano performance, for example, movements of the torso can be used in an expressive manner to convey emotionality in a performance (e.g., Davidson, 2007). In marimba performance specifically, torso movements were found to elicit feelings of happiness, sadness, and anger in raters viewing silent performance videos (e.g., Dahl and Friberg, 2007). In alternate rhythmic contexts, such as dance, torso movements are also critical for emotional communication (e.g., Wild et al., 2017). Overall, the postural sway cluster may reflect an emotionally expressive approach, and therefore was considered to encompass an emotionally vibrant style. Interestingly, such emotional intention may have been completely absent in the energy efficient performance cluster.

The energy efficient cluster may prioritize efficient movements aimed at reducing potential performance-related injuries. This cluster was represented by movements of the upper body and torso occurring along the mediolateral (i.e., left to right) movement axis. It is hypothesized that performers demonstrating this style employed the most direct path along the instrument to position the body and upper limbs in position to play the correct notes (see also Broughton and Stevens, 2009). Such an approach may reflect a style that emphasizes economical movement to either conserve energy or to prevent injury (e.g., Brandfonbrener, 2003; Ackerman et al., 2012; Narducci, 2020). This hypothesis may be supported by the fact that all performers in this study were experienced musicians who would be familiar with methods of maximizing energy efficiency while minimizing potential injury. The energy efficient cluster may employ such a style to reduce muscular loads related to biomechanical constraints (e.g., Furuya and Kinoshita, 2008; Beveridge et al., 2020). As a result, the energy efficient performance style may resolve the redundancy problem by executing movements directly related to sound production.

The final cluster was considered to represent a blend of performance styles observed in this study. Performers in this cluster failed to demonstrate clear delineation from any of the alternate clusters across any of the movement dimensions. When considered along with biomechanical constraints and redundancy problem mentioned in the introduction, it may be suggested that performers in this group have yet to solidify a specific performance style, but rather seem to borrow elements of numerous styles. As shown in Figure 1, there are repeating notes and phrases within the performance. Surprisingly, it appears that performers in this group failed to demonstrate a consistent grouping of motion patterns in line with their counterparts in other clusters. As a direction of future pursuit, it would be critical to investigate whether this performance style is experience driven. More specifically, would expert marimba players demonstrate this blend of style more predominantly than novice or trainee marimba players?

Lastly, it is important to consider the effects reported here with previous theoretical positions. In their qualitative study of marimba performance motion, Broughton and Davidson (2016) postulated that performance styles are hierarchically organized (see also Davidson and Correia, 2002). That is, musical styles may operate as a center for expressive movement within a larger hierarchical framework wherein localized movements at various limb segments are altered in line with the performance context. This interpretation is also supported by motor schema theory (e.g., Schmidt, 1975) wherein the execution of movement patterns involves a set of motor commands that are retrieved from memory and adapted to a particular situation (i.e., the musical notes to be performed). The present study agrees with both theories in that the styles identified here may function as a form of performance filter that can be altered and subsequently executed for any specific performance or instrument, as well as in line with the performer’s desire to convey emotional expression. While encouraging to consider, such a hypothesis requires additional systematic investigation, perhaps utilizing a limb orientation approach (i.e., joint angles).

Nevertheless, this study provides some of the first empirical documentation regarding how upper limb kinematics may cluster together to form a performer’s style in marimba performance. This knowledge is pertinent to further understand sensorimotor control in percussion in general and marimba performance specifically. The emergence of four clusters of limb kinematics was surmised to reflect expressiveness, postural sway emotionality, efficient movements, and a blend of styles for marimba performance. Such findings may be leveraged in the future to guide theories of motor learning in music using empirical evidence of motor control underlying performance styles.

## Data Availability Statement

The raw data supporting the conclusions of this article will be made available by the authors, without undue reservation.

## Ethics Statement

The studies involving human participants were reviewed and approved by the University of Toronto Research Ethics Board. The patients/participants provided their written informed consent to participate in this study.

## Author Contributions

TL designed the study, collected the data, co-designed the computational analysis, interpreted the data, and wrote the manuscript. AH secured funding to conduct the study, designed the study, was responsible for participant recruitment and participation, and interpreted the data. TH conducted the computational data analysis and was responsible for co-designing and completing the principal components and cluster analysis, interpreting the findings, designed the figures, and contributed to manuscript composition. MT secured funding to conduct the study, interpreted the data, and wrote the manuscript. All authors contributed to the article and approved the submitted version.

## Funding

This study was funded *via* a Canadian Foundation for Innovation grant awarded to AH and MT.

## Conflict of Interest

The authors declare that the research was conducted in the absence of any commercial or financial relationships that could be construed as a potential conflict of interest.

## Publisher’s Note

All claims expressed in this article are solely those of the authors and do not necessarily represent those of their affiliated organizations, or those of the publisher, the editors and the reviewers. Any product that may be evaluated in this article, or claim that may be made by its manufacturer, is not guaranteed or endorsed by the publisher.
